# The etiology and pathogenesis of type 1 diabetes – A personal, non-systematic review of possible causes, and interventions

**DOI:** 10.3389/fendo.2022.876470

**Published:** 2022-08-25

**Authors:** Karsten Buschard

**Affiliations:** Bartholin Instituttet, Rigshospitalet, Copenhagen, Denmark

**Keywords:** type 1 diabetes, etiology, pathogenesis, virus, beta-cell activity, gluten, sphingolipids, sulfatide

## Abstract

In this review after a lifelong research career, my personal opinion on the development of type 1 diabetes (T1D) from its very start to clinical manifestation will be described. T1D is a disease of an increased intestinal permeability and a reduced pancreas volume. I am convinced that virus might be the initiator and that this virus could persist on strategically significant locations. Furthermore, intake of gluten is important both in foetal life and at later ages. Disturbances in sphingolipid metabolism may also be of crucial importance. During certain stages of T1D, T cells take over resulting in the ultimate destruction of beta cells, which manifests T1D as an autoimmune disease. Several preventive and early treatment strategies are mentioned. All together this review has more new theories than usually, and it might also be more speculative than ordinarily. But without new ideas and theories advancement is difficult, even though everything might not hold true during the continuous discovery of the etiology and pathogenesis of T1D.

## Introduction

Type 1 Diabetes (T1D) has been studied intensively during the last 5 decades to understand, prevent, or even cure the disease. It is established that it is autoimmune and thus at the very end driven by T cells (1). Also, that autoantibodies exist and that these can be used for diagnoses (2). Various attempts have been tried to arrest T1D; these include mainly immune depressing therapies like anti-lymphocyte serum (3) and other immune antibodies (4) as well as antigen treatments with beta-cell autoantigens like Glutamic Acid Decarboxylase (GAD) (5) or insulin (6). Twin studies have shown that about 50% of the T1D disposition is inherent, the rest is of environment reason (7). Other kind of studies are on the same line (8). However, the disease incidence has increased and more than doubled in the last decades (9), meaning that the importance of the environment factors may have increased. As mentioned later in this review, tissue types are the most significant inherent components, but many others exist with association either to the beta cells or to the immune system.

Regarding the etiology, most scientist but not all think that enterovirus are the most likely candidates (10, 11) but also stress of the beta cells must be of importance (12). This settles the scene for this review, hopefully with new ideas and new thoughts.

This is a personal review with which I will explain a complete story of the development of type 1 diabetes (T1D) from the first etiology through the pathogenesis to the establishment of the disease. The review is personal because not everything is proven, and some parts are theoretical (T). This will be mentioned as just indicated. However, references will be used as much as possible.

What are the initiating factors of T1D? I am convinced that virus, most likely enterovirus, is involved for several reasons. Very good animal studies exist to prove this, and here I should mention the EncephaloMyoCarditis (EMC) M-strain virus model. In 1976 (13) and 1983 (14) we showed that healthy BALB/c mice within a week develop diabetes in about 1/3 of the animal cases. This most likely depended on the T cell immune system since nude mice (13, 14), antiglobulin-treated mice (15), and BALB/c ByJ mice given anti-T-cell antibodies (16), did not develop diabetes. Thus, these models were dependent on the T cells just like in human T1D. Several other strains of the virus have been used. The EMC-D strain also lead to development of diabetes but this was toxic by itself against the beta cells, like poliovirus against neural cells, and not relying on the immune system (17); it more resembled the toxic diabetes seen nearly exclusively in South Korea and Japan. However, the vast majority of humans with T1D are dependent on the immune system, the reason why immune suppressive treatment, such as an anti-lymphocyte immunoglobulin can be used with beneficial effect (18). In humans there are good arguments for virus involvement: T1D is most commonly seen in the months of autumn during which enterovirus infections are most frequent, and there are several indications showing presence of virus especially in stool (10, 19, 20). In recent years samples and biopsies from new human T1D cases have also shown footprints of virus infections in islet tissue (21). On the other hand, virus is not present in significant amounts in human patients. Although another study disagrees (22), it has been found that there is a 37% lower risk of T1D in youngsters after vaccination against rotavirus (23). This is not a final proof of the involvement of virus but a strong argument that enterovirus might be involved in the pathogenesis of T1D.

**Figure 1:**
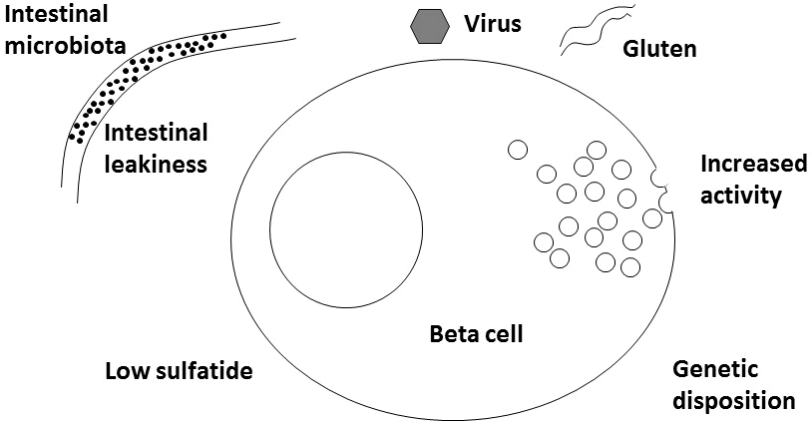
Schematic drawing of various factors involved in development of Type 1 Diabetes.

A new player for virus might be interferon alpha. Either induced by the virus infection or given therapeutically for example against melanoma or hepatitis C (24, 25), interferon alpha can induce 2,5-synthetase (26) and RNAseL that in turn can fight virus but also RNA from the host cells themselves. mRNA from these compounds have been found increased in islets from newly diagnosed human T1D patients (27). The significance of this is supported by gene polymorphisms between several genes in this cascade and T1D (27). Interestingly, interferon alpha responsiveness is increased in whole blood cells from patients with T1D and from NOD mice also (28). In beta cells 2,5-synthetase is substantially more common than in alpha cells which may add an explanation to the fact that only beta cells are hurt and not alpha cells (26). Thus, it is a possibility (T) that virus infection in a beta cell acts indirectly as described by injuring RNA of various enzymes, including the sphingolipid involved ones (see later), and thereby commence the development of T1D (**Figure 1**).

## The intestine

Regarding the microbiota*, Akkermansia muciniphila* is known to be present at a lower amount at the diagnosis of T1D (29). Further, more *Bacteroides* are seen at diagnosis of T1D and this is supposedly not an advantage (30); abundance of Bacteroides is associated with less production of butyrate which is protective in the pathogenesis of T1D (31). It is likely that the intestinal bacterial flora directly or through endotoxins influences L cells that produce GLP-1 and GLP-2 (32). This is important as it offers an explanation for the interaction between the intestinal flora, glycaemic control, and body mass index (32). Also, virus can influence the intestinal flora; there is a connection between enterovirus and dysbiosis of bacteria (33).

A main hallmark of T1D is the increased intestinal permeability. This has been described using the method of absorption of the lactulose:mannitol ratio. It revealed that the permeability is increased in newly diagnosed T1D patients (34). This has been demonstrated also in patients having diabetes for more than 4 years as well as in a subject with pre-T1D or antibody-positive patients (34). It is not known whether such a potential patient has an increased permeable intestinal barrier function even before he becomes antibody positive. This might be a permanent characteristic of that person. In favor of this hypothesis, first degree relatives also have increased intestinal permeability compared to controls (35). Several environmental factors are known to affect the permeability. *(1) Enteroviruses*. Enteroviruses *per se* effect the enterocytes (36). However, it is difficult to imagine that an enterovirus infection persists during decades so this alone might not be the cause of increased permeability through many years, as just described. *(2) Akkermansia*. *A. muciniphila* is known to decrease the permeability of the intestine which happen through an interesting mechanism; the *Akkermansia* bacteria is degrading the mucus layer close to the enterocytes but the result is that more mucin is produced and the intestinal barrier is tightened (37). *(3) Gluten*. Intake of gluten is known to increase the permeability (38). *(4) CXCR3 ligands*. These include various chemokines released in the intestinal environment which through the upregulation and MyD88-dependent zonulin will increase the permeability (35). *(5) Serine palmitoyltransferase.* Sphingolipid *de novo* biosynthesis in which serine palmitoyltransferase is important for the intestinal barrier function (39). Thus, it must be concluded that T1D is a disease of a relatively leaky intestine and that several factors might contribute to this. Apart from permeability and intestinal flora, compartments seem to be of importance. In a human study of fecal transplantation from colon to duodenum, autolog Tx showed significantly better results than allogen Tx on glycemic values among recent T1D patients (40). This interesting area must be developed further in the coming years.

## Beta-cell activity

Hyperactivity of insulin secretion has been found in patients with prediabetes (41). This may *per se* give more ER-stress which in principle might increase the risk of odd insulin molecules that potentially might be immunogenic; these include wrongly spliced insulin and deamidated glutamin insulin (12). Furthermore, the volume of pancreas has been found substantially lowered in patients with T1D; in relation to body weight, pancreas volume was only 55% of the control values, whereas in patients with type 2 diabetes (T2D) it was not significantly reduced (42). Also, pre-T1D patients with disease-associated autoantibodies showed lower pancreas weights; when having one autoantibody they displayed 75% of the control value (43). Whether also healthy family members have a lower pancreas volume is not known for sure. Since the islets constitute only about 1% of the pancreas volume, it is obviously the exocrine pancreas is reduced during T1D but also the number of islets might be affected (44). Due to these data, it must be considered as part of the T1D pathogenesis to have a lower exocrine pancreatic volume. The reason might be related to the affected nerve stimulation of the organs as discussed below in relation to the sphingolipids.

Another explanation for the low pancreas weight should also be mentioned (T): A few generations ago, before intake of refined sugar products became common, the main stress factor for beta cells was high amounts of fat, which uptake into the cells depends on insulin. A diet containing a high percentage of fat requires high quantities of lipase and consequently high activity of the exocrine pancreas. Thus, the endocrine and exocrine function are interdependent. Interestingly, individuals that are prone to develop T2D are often pyknic individuals, who as arctic people may have experienced a cold climate which have led them to eat a high fat diet. In contrast, individuals who develop T1D are often slim and tall with no tradition for gannet food. Thus, these individuals may likely have a small pancreas, hence a small beta-cell mass combined with a higher intake of refined sugar products which might lead to beta-cell stress and higher risk of T1D.

Lower beta-cell volume should be more stressed by intake of Western food. However, lack of rest for the beta cells could be more important than experience of stress (T). Hence, intake of food each other day only, reduces the incidence of diabetes in NOD mice (45).

## Sphingolipid

Glycosphingolipids are supposed to play an important role in the metabolism and function of the beta cells (46). In many ways, beta cells resemble neural cells. This is supported by novel findings that co-transplantation of human pancreatic islets with neural stem cells increases beta-cell proliferation and vascular regrowth (47). Also, during the foetal development of beta cells, stem cells from neural ectoderm are found *in situ* at the beta cells (48). Supposedly these neural cells somehow influence the beta cells. Very likely, this could also be the case regarding sphingolipids including sulfatide (49); these compounds are intensively present in the myelin tissue surrounding the nerves, having the function of facilitating electric impulses (50). In beta cells, especially sulfatide is important. It facilitates folding of proinsulin and insulin. This process seems to be important since in newly diagnosed T1D patients, the amount of sulfatide is substantially lower as later discussed. In these patients, there is an accumulation of proinsulin with an increased proinsulin to insulin ratio (51), and up to 20% of the proinsulin molecules are misfolded (52). Also, at low pH sulfatide preserves the insulin crystals (53, 54). Sulfatide facilitates exocytosis and afterwards opens the potassium channels so that the individual beta cells can rest to build up new secretory granules close to the cell membrane, meanwhile other beta cells take over the secretory responsibilities (55, 56). Furthermore, sulfatide inhibits cytokine secretion (57), it is antagonistic to TLR4 (58), and also, sulfatide may hinder or reduce the amount of TLR4 molecules at target cells for LPS (59), and it facilitates NKT cells after presentation by CD1 molecules (60, 61); in other words, sulfatide has anti-inflammatory effects. Regarding insulin resistance, the level of sulfatide in the blood is highly inverse correlated to this (62).

For normal production of sulfatide, serine uptake is important. First, serine is used to synthesize ceramide, hereafter beta-Galactosyl-Ceramide (beta-GalCer), and finally sulfatide. All the enzyme systems are well known (63). If, for some reason, serine is not bound in sufficient amounts to its receptor, alanine and glycine can take over, but then deoxy sphingolipids are synthesized (64). Physiologically, these compounds do not act the same way as sulfatide in the beta cell, and furthermore they are potentially apoptotic (65). In neural cells, it has been described that lowering plasma deoxy sphingolipids by oral L-serine supplement alleviates neuropathy in diabetic rats (66). In T2D patients low plasma serine and high deoxydihydroceramide are associated with diabetic neuropathy (67). In NOD mice treatment with L-serine in the drinking water reduced the diabetes incidence from 71% to 43% (68). Interestingly, during progression of human T1D, the serine levels in peripheral blood has been found to decrease (69).

In humans at diagnosis of T1D, we have found that in the islets, the amount of sulfatide is only 23% of that seen in non-diabetic controls (70). In a new study from the TEDDY group, low plasma levels of sphingomyelin are correlated to development of islets autoimmunity (71). Furthermore, we have found that several but not all enzymes involved in the sulfatide trail measured in the islets at mRNA level, have been downgraded at T1D diagnosis (70). When co-factors are considered it could be more than 50% lower activity of a specific enzyme. This can explain the anticipated higher deoxy sphingolipid and ceramide levels in pre-diabetic islets (T) to T1D in the genes of several enzymes involved in the sphingolipid metabolism; the odds risk for having T1D compared to control persons is up to 1.47 (70).

Another possible explanation for the presence of deoxy sphingolipids in the islets is suggested (T). These could also be provided by neural cells in the islets that are damaged by neuropathy and which are present in relatively high amounts (72). This fits well with the fact that deoxy sphingolipids have been found in neural cells (73) and that neural cells in the islets can be conserved by treatment of fenofibrate (72). Interestingly, the type A enteroviruses actually seem to be more frequent in healthy persons than the type B (74). The type A viruses are more associated with neurological manifestations whereas the type B viruses are more associated with gastrointestinal manifestations. In the case of T1D (T) this might be due to infection of both Coxsackie A and B strains maybe more or less simultaneously (19).

The changes in the sphingolipid enzymes in the islets, which are seen at diagnosis of T1D simultaneously with a state of anergy for at least one third of the beta cells (75), might have a normal physiological reference in fasting or even hibernation. For this function, it might be imagined that the potassium channels are opened and stay as such (T). This could happen if sulfatide in the channels is not broken down which might be the case if arylsulfatase is reduced as it actually is in islets of newly diagnosed T1D patients (70). Also, it might be anticipated that sulf-lac-cer could be active in opening the potassium channels, maybe more than sulfatide as a defence of nature for preserving a vulnerable beta cell in danger (T). This is likely the case in T2D in which sulf-lac-cer can be detected in peripheral blood (62). Interestingly, the enzyme pathway from ceramide to lactosyl-ceramide is the only one which is upregulated in the newly T1D islets (70).

Beta cells seems not to be 100% synchronized. It may be that only some of the cells is anergic (70), which have the consequence that the non-anergic ones are hyperactive and thus more vulnerable. Due to creation of defective ribosomal products (DRiPs, see later) these cells might be victims of attack from the immune system whereas at the same time the anergic beta cells might be victims from apoptosis or necrosis. Thus, synchronized action of all or most of the beta cells to active state and not letting the minority of them be hyperactive, must be a goal by itself. Furthermore, the beta cells might not be completely identical and could react differently (76).

An interesting possibility should be mentioned regarding how the amount of sulfatide could be influenced (T). Children may have sulfatide antibodies. It may be that these antibodies also react against sulf-lac-cer, which the Sulph I antibody from mice actually does (77). It could then be (T) that sulf-lac-cer opens potassium channels which might be the reason why foetal beta cells to a large extent do not secrete insulin. It might be the same mechanism that could be in play when animals hibernate (T). Antibodies against sulf-lac-cer could remove this molecule resulting in normally functioning beta cells. This could explain why foetal beta cells might not be replaced in newborns but only change properties. These antibodies will not hurt the secreted sulfatide since this is degraded to gal-cer which is then re-up taken (49). That insulin is secreted in children of diabetic mothers during foetal life, could be explained by antibodies from the mother.

Why is it that small kids to high extend develop antibodies against a self-molecule? This could be due to intake of various food items especially milk which contain mycobacteria (78). Mycobacteria are used in Freud’s adjuvant and are known to have a lipid wall which contain very long fatty acids with 60-80 carbon atoms. But, interestingly, they also have a content of sulfatide. It can be imagined that these immune enhancing bacillus through their content of sulfatide can mediate production of anti-sulfatide antibodies. Mycobacteria are seen in milk, cheese and other items related to animals. In Sardinia having a very high T1D incidence, a special Sada goat exists, and milk and cheese from goats have a decent number of mycobacteria (79). It is obvious that the mycobacteria in play cannot be directly pathogenetic in humans, in contrast to *Mycobacterium tuberculosis* or *Mycobacterium lepra* for which diseases anti-sulfatide antibodies are common (80). After phagocytosis of mycobacteria, phagosome-lysosome fusion in macrophages is prevented which enhances the immunogenicity of sulfatide (81).

## Intake of gluten

When the intestinal barrier is increasingly open as it is in (pre)T1D, gliadin product from gluten could enter the bloodstream (82) and create a (more) inflammatory environment in the intestinal compartment and in the islets. Why is this important? Celiac disease is closely related to T1D and is a condition of intolerance to gluten, especially gliadin. There is co-morbidity of celiac disease and T1D; thus, about 10% of patients with T1D also have celiac disease. Interestingly, in far the most of these cases T1D is the first of these two diseases to appear (83), which could argue that if celiac disease develops first, then the patient shifts to gluten-free (GF) diet and seldomly T1D will develop. The first indication of the importance of GF diet in T1D was found in 1999 when we fed NOD mice a GF diet and discovered a much lower incidence of diabetes than for the control mice (15% versus 64%) (84). This has been confirmed in many other studies both in NOD mice and BB rats (44, 85). Even the cultivars of wheat seem to play a role as it has been found that modern western wheat is the most diabetogenic (86). Highly interestingly, recently it has been published that there is an association between gluten and risk of islet autoimmunity or T1D in humans (87). Thus, even in the earliest state of T1D development, intake of gluten seems to be of relevance. In the study, 5500 children with an increased genetic risk for T1D were chosen from a Finish birth cohort, and during a 6 years period the gluten containing food items were recorded. The results showed that high intake of gluten increased the risk of both islet autoimmunity and T1D in the magnitude of factor two compared to low or no intake of gluten (87).

What is the mechanism behind the connection between gluten intake and T1D? GF diet changes the intestinal microbiota and the proportion of *Akkermansia muciniphila* bacteria is increased (88). This bacterium adheres to enterocytes and strengthens the integrity of the intestinal barrier thus it is less penetrable (89). On the other hand, gluten containing diet induces the presence of transTissueGlutaminase (tTG) in the intestinal wall, which facilitates deamidation of various proteins e.g. converting glutamine to glutamate. Due to the increased permeability of the intestine, even relatively large fragments of gliadin molecules can be absorbed and, indeed, we have found a 33-mer gluten peptide present in the islets (82). At this location, this molecule can again facilitate tTG upraise which is important for the creation of DRiPs (90). DRiPs are effectively loaded to HLA-DQ2 and by presentation in the regional lymph nodes, a T cell immune reaction may come into play. Furthermore, gliadin fragments facilitate higher activity of dendritic cells (91), NK cells (92) and CD4+ T-cells (44) which increase the degree of inflammation. Interestingly, the 33-mer gliadin molecule has been described to perform oligomerization which might further attract immune cells (93).

Also, GF diet during pregnancy has been studied in NOD mice. We have found that intake of no gluten from conception to delivery dramatically lowered the diabetes incidence in NOD mice from 63% to 8% (94) together with lowering insulitis (44). This has been confirmed in a human epidemiological study with more than 60.000 participants (95). Here it has been found that the offspring of the participants with the 10% lowest gluten intake, have a two-fold reduced risk of developing diabetes compared to the offspring of the 10% of the participants with the highest gluten intake. What could be the reason for this? A study from Finland has found that about 80% of normal persons display CD4+ T cell proliferation responses against gliadin (96). Interestingly, this was seen to a lower degree and only in 50% of patients who were antibody-positive (preT1D) or were newly diagnosed T1D patients. After some years, this gliadin reaction was normalized again to the level of the background population. The explanation (T) for the pregnancy studies could be that when gliadin is not experienced in the foetal life, then the likelihood for a T cell reaction against wheat might be larger and if so, gliadin fragments should not be able to reach the pancreas through the bloodstream. The lower reaction against wheat in newly diagnosed T1D patients could be due to the opposite effect combined with the fact that these patients have a relative open intestinal barrier making access of gliadin peptide easier (T).

Also, in later stages of T1D development and even after diagnoses, gluten intake plays a role. Thus, newly diagnosed T1D children showed effect of a GF diet, as these displayed a better remission after one year; two of the GF-patients were even out of insulin during longer periods (97). Most recently, a 12-month intervention trial from Prague regarding intake of gluten in recent-onset T1D patients has been performed (98) in which, forty six children (mean age of 10 years) were divided into a GF or a control group. After 12 months the gluten-free patients displayed better HbA1c and received less insulin (98). However, as T1D is a troublesome disease to be diagnosed with, and it will be even harder to manage a simultaneous GF diet as well. Therefore, we have suggested intranasal exposure to gliadin which lowers the diabetes incident in NOD mice (99). This has not as yet been tried in humans but interestingly, bakers who are exposed to gliadin during their daily work and for whom gliadin can be isolated from the nasal mucosa, only 57% of the expected cases developed T1D compared to the background population (100). Whether GF diet will work for a person with pre-T1D by delaying or preventing the clinical disease must be investigated as soon as possible.

In the context of the beneficial effect of GF diet, we investigated the effect of intake of an excessive amount of gluten in NOD mice and expected an increase in diabetes incidence. In contrast, we found that the excess of gluten dramatically lowered the incidence of diabetes (101). How could that be? It has been found that gluten-reduced diet is accompanied with reduction in the butyrate producing *E. halli* and *A. hadrus* (102). Opposite, a study in mice has found that gliadin increases the levels of *Eubacterium* and *Dorea* species which contributes to the butyrate production (103). It is established that butyrate produced by the intestine can lower the incidences of diabetes in NOD mice (104). Butyrate boosts the number and function of regulatory cells (104, 105), and it enhances the gut integrity and decrease the diabetic cytokines such as IL-21 (104, 105). This may be seen in connection with other short-fatty acids which also have anti-diabetic effects. A diet both being GF and having a good production of butyrate should be developed.

## The actual destruction of beta cells

Until now in this review the induction of the disease has been described, but how is the destruction of beta cells actually happening? Before this, close to the diagnosis of T1D, a kind of anergy for the beta cells takes place. Initially, T cell reaction is slightly ongoing but at this stage the beta cells mainly look normal and a high percentage are alive and in principle able to function (75). However, clinically earlier in some patient but at the latest at the end of the remission period, the disease process accelerates, and new cellular players may come into account or at least be active to a higher degree than seen before. The cells that are involved are macrophages (106), dendritic cells (107), B lymphocytes (108), all having an important role in antigen presentation. This may take place in the regional lymph nodes, and T cells interaction is becoming more and more critical. Before this, cells from the innate immune system like NK cells seem to be involved (109).

When a significant part of the beta cells are anergic and some destroyed, then the remaining must work really hard, and maybe harder than ever because of simultaneous insulin resistance which is seen during T1D development (110). As mentioned this is correlated to low levels of sulfatide (58). The mechanism might be that sulfatide blocks TLR4, stimulation of which mediates insulin resistance (111).

What is actually destroying the beta cells? Mainly due to research in the animal models, BB rats and NOD mice, it was proposed in 1985 by Bendtzen et al. that cytokines, especially IL-1β or the mixture of IL-1β, TNF-α and IFNγ may be mediators of beta cell deterioration (112). Indeed, this can be shown for isolated islets *in vitro*, namely that beta cells exposed to such a cytokines mixture are destroyed in a necrotic process. Signs of this can be seen in both BB rats (113) and especially NOD mice (114). However, human islets are less sensitive to cytokine destruction and, furthermore, in human samples of newly diagnosed T1D patients from the DIVID and nPOD study, cytokines cannot be found, except minor concentrations of IFNγ (75). What is then responsible for the destruction of the beta cells? Supposedly, perforin is active in cell destruction (115). This is a well-known molecule secreted during inflammatory processes which follow the described stages of beta-cell anergy.

The cytotoxic T cells that sooner or later are created in the inflammatory process can be delayed or stopped by Tregs. In 1980, we were the first to describe in a functional study, that there is less suppressor or regulator cell activity in newly diagnosed T1D patients (116). This has later been confirmed by many others. Also, the amount of regulatory FoxP3 cells have been examined but several studies indicate it is more the function than the number of regulatory cells that is the problem for the T1D patients (117).

All autoimmune diseases are driven by T lymphocytes. Why is it then that among the first cells to arrive in the insulitis lesion are cells from the innate immune system? These cells cannot create autoimmunity, but they are usually present due to virus or other infections. Highly interesting in this context is that the T cells alone cannot start beta-cell autoimmunity (T). This statement is supported by several findings: i) When a person has only one beta-cell antibody, seldomly he will have clinically diabetes although T cells have been involved in creating the antibody; ii) It is not possible in animal models to immunize clinical diabetes e.g. by using (allogeneic) islet cell tissue and Freunds adjuvants (118); iii) Treatment with insulin to psychiatric otherwise normal patients in order to give insulin shock, did not result in clinical diabetes despite that the insulin in the 1950ties were dirty and improper and must have resulted in development of insulin antibodies (119). Thus, the T cells cannot do the work alone and something else must be involved. Most likely it may be the innate immune system e.g. NK cells being attract by microbial agents or by an experience of tumour-like cells in in the islets or surroundings. However, then the T cells arrive later, and the process reaches a point of no return. The T cells developed phylogenetically simultaneously with the mammals as they became warm blooded. Their main function is to fight cancer cells which are more common than in coldblooded reptiles. Consequently, the T cells “may believe” that the beta cells in the beginning of insulitis are tumour cells, which should be destroyed. Normally, the beta cells are covered by sulfatide which is an anti-inflammatory molecule and at least as investigated in animal models, it is more present during foetal life. Actually, the adaptive immune system has not learnt to accept the beta cells without sulfatide. Sulfatide is relative less present in active beta cells (120) as seen in foetuses of diabetic mothers. The foetuses may then better signal “self” to the immune system. Later in life these children have only less than half the risk of getting T1D compared to kids of diabetic fathers. We have found that neonatal stimulation of beta cells by arginine or other beta cell stimuli reduces the later diabetes incidence in BB rats (121). In this study, we suggested a tolerance mechanism and most recently, it has been shown that CD4+ T cells from cord blood of offspring of diabetic mothers have a reduced response to pro-insulin and insulin compared to controls as well as to children of diabetic fathers who have more than a double risk for developing T1D (122).

What is to do for causal treatment at diagnoses? Sulfatide needs to be replenished at the surface of the beta cells. Then the beta cells need to be activated for insulin production which at first may attract the T cells. Therefore, it might be a good idea to inhibit the T cells shortly (days or weeks) either by antibodies fx *anti-CD4 mAb*, or by *steroid* treatment. Simultaneously, but for a much longer time (maybe years), compounds should be given that increases the amount of sulfatide, which could be *serine* or *fenofibrate*. Regarding fenofibrate, which is a PPARα agonist (72), treatment with this have until now in one case of a newly diagnosed T1D patient given freedom of insulin injections ongoing for 30 month (123). Furthermore, the beta cells should not be stressed, and *gluten-free diet* should be initiated as being anti-inflammatory and especially having its effect on the innate immune system.

Regarding genetics, far the most important polymorphisms are due to tissue types which was firstly described by Singal and Blajchman in 1973 (124), and in 1976 by the same group also for class II tissue types (125). Apart from being disposing, a new practical function HLA has been settled. Newly diagnosed T1D patients with the tissue type of HLA-DQ2 can be treated successfully with GAD in inguinal lymph nodes in order to delay their disease (126). At least about 50 other polymorphisms have been demonstrated either relating to beta cells or the immune system. Among the first ones are eight polymorphic genes related to the sphingolipid metabolism. However as outlined above, environmental factors not least viruses seem to be crucial for the development of T1D.

## Final remarks

Based on studies, new speculations, and ideas my conclusive remarks should be that I have tried to give a personal, complete story of how T1D initially starts and how it ends up as an autoimmune T cell driven disease. It is my hope that at least some of my ideas and suggestions will receive an adequate validation, so that T1D can be prevented or treated, likely not completely but to the incidences levels that exist in the 1950s which is the same as the incidence levels in Russian Karelia today (127), in contrast to the five times higher T1D pressure in the neighboring Finnish Karelia being much more modernized and westernized.

## Author contributions

The author confirms being the sole contributor of this work and has approved it for publication.

## Conflict of interest

The author declares that the research was conducted in the absence of any commercial or financial relationships that could be construed as a potential conflict of interest.

## Publisher’s note

All claims expressed in this article are solely those of the author/s and do not necessarily represent those of his affiliated organizations, or those of the publisher, the editors and the reviewers. Any product that may be evaluated in this article, or claim that may be made by its manufacturer, is not guaranteed or endorsed by the publisher.
